# Feeding-Related Knowledge, Attitudes, and Practices among Grandparents in Singapore

**DOI:** 10.3390/nu11071696

**Published:** 2019-07-23

**Authors:** Bernadette Q.M. Tan, Jia Min Hee, Ka Shing Yow, Xueling Sim, Miho Asano, Mary Foong-Fong Chong

**Affiliations:** 1 Ministry of Health Holdings, Singapore S099253, Singapore; 2 Yong Loo Lin School of Medicine, Singapore S117597, Singapore; 3 Saw Swee Hock School of Public Health, National University of Singapore and National University Health System, Singapore S117549, Singapore

**Keywords:** grandparent, feeding practices, grandchild, food knowledge, practice, food handling

## Abstract

Childhood obesity is a growing concern worldwide. Though multifactorial, the family environment exerts significant influence on children’s eating habits. Grandparents are increasingly involved as caregivers and they can significantly influence their grandchildren’s eating habits. Yet, literature on this topic is lacking. This exploratory sequential mixed methods study (qualitative interview and interviewer-administered questionnaire) aims to understand grandparents’ knowledge, attitudes, and practices on the feeding of their grandchildren in Singapore. A total of 11 interview participants and 396 questionnaire respondents with at least one grandchild, aged 12 years and below were included. Qualitative interviews informed the questionnaire development. Responses to interview questions about knowledge, attitudes, and practices revealed sub-themes such as knowledge on the impact of feeding, attitude toward feeding role, and challenges to feeding. Of the 396 participants, 35% were primary caregivers (defined as the person who spends the most time with the grandchild and performs most of the caregiving tasks). Nutritional knowledge was fair (median score 5/8), with misconceptions centered around healthy feeding practices. Grandparents who were primary caregivers, female, Malay, and younger than 70 years old believed that they played an important role in feeding their grandchild (*p* < 0.05). Overall, 47.2% of the grandparents rarely or never set a maximum limit on the amount of unhealthy food eaten, of which 77.1% are non-primary caregivers. In comparison, primary caregivers tend to set a maximum limit to the amount of unhealthy food their grandchildren eat and choose a wide variety of food (*p* < 0.05). These findings support the need for further improvement of grandparents’ feeding knowledge and practices as part of tackling childhood obesity.

## 1. Introduction

Childhood obesity is an increasingly prevalent problem worldwide, presenting itself as a major public health challenge of the 21st century [1]. According to the World Health Organisation [2], there were 41 million obese children in 2016 and this is predicted to increase to 70 million by 2025. Singapore has seen a significant increase in its childhood obesity rate over the years. Among students aged 12 years old, the obesity rate has increased from 2.2% in 1976 to 15.9% in 2006 [3]. Obese children are more likely to be obese in adulthood, predisposing them to diseases such as diabetes mellitus, hypertension, and other cardiovascular complications [4].

Diet plays a key role in contributing to weight gain and obesity [5]. Family and the home environment are well-established sources of influence on children’s eating habits, especially in early and middle childhood [6]. While poor parental feeding practices such as using unhealthy food to reward desirable child behavior [7] and overfeeding [8] can adversely affect children’s dietary behaviors and their health, there is growing evidence to suggest that grandparents play an increasingly important role in influencing their grandchildren’s feeding practices [8,9,10,11,12,13,14,15,16].

Previous studies conducted in China [8,9,10], Japan [11,12], Australia [13,14], the United States [15], and the United Kingdom [16] have established that grandparents play a significant role in shaping their grandchildren’s feeding practices. Certain recurring themes related to knowledge, attitudes, and practices were common across the studies. In terms of knowledge, the Asian studies showed that grandparents tended to harbor various misconceptions about healthy eating [8,10,11] including the belief that an overweight child is evidence of successful feeding [8,10]. Regarding attitudes, the Australian, American, and the United Kingdom studies found that grandparents took pride in their feeding role and believed that they had the authority to allow their grandchildren to indulge in less healthy food [13,14,15,16]. With regard to practices, grandparents generally expressed their influences over their grandchildren’s diet through food preparation and determining the quantity of food consumed [8,9,10,11,12,13,14,15,16]. In a study in Japan by Morita in 2019 [12], grandparents’ residence was found to be associated with an increased risk of free snacking behaviors in the first grade but negatively associated with the body mass index scores in the second grade after adjustment for family sociodemographic and obesogenic behaviors. It is unclear if these findings are applicable, in a local context, in Singapore as feeding practices are often strongly influenced by local cultural and social factors [17,18].

A study investigating the various roles that grandparents play in the caregiving of their grandchildren found that grandparents in Singapore are increasingly involved in the care of their grandchildren, including preparing and cooking their meals [19]. According to the National Survey of Senior Citizens 2011, 29% of elderly aged between 65 and 74 years were involved in the caregiving of their grandchildren [20]. This proportion is expected to rise given the steady increase of dual-income families [21] and state-driven policies [22,23,24].

The aim of this exploratory study was to understand grandparents’ knowledge, attitudes, and practices (KAP) on the feeding of their grandchildren in Singapore. This may provide insights into feeding practices of grandparents, which may be contributing to the current childhood obesity epidemic.

## 2. Materials and Methods

### 2.1. Study Design

An exploratory sequential mixed methods [25] study was conducted from June 2017 to February 2018. The initial semi-structured qualitative interviews were used to guide the development of the quantitative interviewer-administered questionnaire. The interviews explored grandparents’ perceptions about healthy eating and their role in influencing their grandchildren’s eating habits. The questionnaire was a cross-sectional study that assessed feeding KAP among grandparents. All subjects gave their informed verbal consent for inclusion before they participated in the study. Ethics approval was obtained from the National University of Singapore Institutional Review Board (S-17-272).

The study’s target population was grandparents who interact significantly with their grandchildren of up to 12 years of age. Participants fulfilling the following inclusion criteria were recruited: (1) able to understand and respond to the questionnaire (in English, Mandarin, Tamil, Malay, or dialect); (2) is a Singapore Citizen or Permanent Resident of Singapore; (3) has at least one grandchild not more than 12 years of age; (4) physically spends time with that grandchild for at least two days a month, regardless of the time spent each day. For a qualitative interview, an additional inclusion criterion was agreement to audio-recording of the interview.

The criterion set for the age of the grandchild was justified by the finding that eating habits of children up till 12 years old are significantly influenced by the family [6]. The criterion set for the minimum amount of interaction between the grandparent and grandchild was based on a similar American study [15]. As it was not specified whether two full days of interaction time between the grandparent and grandchild were used as part of the inclusion criteria in the American study and given the lack of similar studies, it was arbitrarily decided to disregard the time spent for each of the two days in this study.

### 2.2. Qualitative Methods

#### 2.2.1. Instrument Development

From October to November 2017, face-to-face semi-structured qualitative interviews were conducted with the aid of an interview guide (Table 1). The qualitative interview guide was created by adapting from existing questionnaires [26] and qualitative interview guides [8,9,15,27,28] on parents’ and grandparents’ feeding practices. Questions used in these instruments and results from previous studies [8,9,11,13,15] generated three recurring themes of “knowledge,” “attitudes,” and “practices.” The main questions were primarily designed to be open-ended with the use of clarification questions and probes when necessary to elicit further details. The English interview guide was subsequently translated into Mandarin, Malay, and Tamil (the other non-English languages spoken in Singapore) to facilitate the interview process for non-English speaking participants.

#### 2.2.2. Participant Recruitment and Procedure

Participants who fulfilled the above inclusion criteria were recruited for interviews via convenience sampling method through word-of-mouth recruitment within students of the university. Recruitment ended when no new sub-themes were identified in further interviews. Prior to the interviews, participants were briefed by trained interviewers from the research team about the study using a participant information sheet before verbally giving their consent. All interviews were conducted by trained interviewers in a language understandable by the participants. On average, a single session lasted 20–30 min. Notes on the main points mentioned by the participants were taken and checked back with the participant at the end of the interview. Participants were allowed to clarify any mistakes in the notes and add on to points mentioned. Each interview was audio-recorded and transcribed verbatim [29]. Non-English transcripts were translated to English by team members proficient in the language. Audio-transcript data check was performed by another group of team members not directly involved in the interviews to ensure accuracy of all the transcripts [29]. 

#### 2.2.3. Data Analysis

The Braun and Clarke’s approach [29] was employed for the thematic analysis. Six researchers initially carried out independent manual thematic analyses of four transcripts. Emerging codes were identified from transcripts via line-by-line coding based on the recurring ideas that different grandparents spoke of in different interviews. The coded transcripts were discussed, and a common coding framework and codebook was agreed upon. The six researchers carried out independent thematic analyses of the remaining transcripts using the codebook with new codes discussed online and face-to-face at authors’ meetings. Sub-themes and themes were then identified from the categorization of these codes. After several group discussions between the researchers, the codes and sub-themes were further refined, and the final analytical categories were established. The emerging codes and sub-themes were used in the development of the quantitative questionnaire. Codes were used to generate questions based on either adaptation from existing questionnaires or creation where there were none existing. Categorization of codes into sub-themes facilitated the delineation of each question’s role in the questionnaire.

### 2.3. Quantitative Methods

#### 2.3.1. Instrument Development

Existing validated questionnaires [26] mostly went into great detail about multiple aspects of grandparental and parental feeding practices with little coverage on knowledge and attitudes. Given the scope and exploratory nature of this study, they were not found to be suitable for this study. Thus, with the aid of codes and sub-themes from the qualitative interview component, in addition to referencing existing questionnaires (Table 2), a questionnaire was developed (Supplementary file 1). The themes of “knowledge,” “attitudes,” and “practices” formed the three main categories of the questionnaire. Based on the main codes and sub-themes that arose from the qualitative interviews, it was decided that questions regarding those topics were to be included in the questionnaire. Questions from existing questionnaires were adapted and modified and new questions were created when there were none existing. Responses from the interview participants also formed some of the options for certain questions.

The interviewer-administered questionnaire consisted of 36 questions in five main sections: (1) participants’ demographics, including whether they were the primary caregivers of their grandchildren, defined as the person who spends the most time with the grandchild and performs most of the caregiving tasks [30]; (2) frequency of direct feeding practices (all the time/sometimes/rarely/never) and indirect feeding practices in the form of a multiple response question; (3) attitudes toward own feeding role and toward other caregivers’ feeding practices; (4) knowledge of healthy feeding practices; and (5) challenges and factors influencing feeding practices.

Due to the exploratory nature of the study, the questionnaire was not fully validated. However, the questionnaire underwent several steps in the validation process. Face validity was first established through critical evaluation by experts from Saw Swee Hock School of Public Health.

This interviewer-administered questionnaire was then piloted among the grandparents of the team members who fulfilled the inclusion criteria. Pilot data collected was cleaned. Based on the analysis of this data, the questionnaire was then revised accordingly. This included rephrasing and adding definitions to terms in some questions to improve clarity, re-ordering questions to improve the flow of the questionnaire, combining questions and removing ambiguous questions to shorten the questionnaire, and adding more options to the multiple response questions based on feedback. The final English questionnaire was then translated into Mandarin, Malay, and Tamil.

Cronbach’s alpha was not applied in view of the exploratory nature of the study and diverse nature of food knowledge, attitudes, and practices within each targeted construct. 

#### 2.3.2. Participant Recruitment

As there were no previous studies on the prevalence of grandparents adopting a set of knowledge, attitudes, and practices toward influencing their grandchildren’s feeding habits, a simple sample size calculation for a prevalence study was adopted. For a prevalence (proportion of the population surveyed fulfilling the study’s inclusion criteria) of 50%, at 95% confidence interval and 5% margin of error (e) for the sample population, it was calculated that the sample size required for this study is 385 (based on the following formula) [31].

$$N=\left[\frac{Z^{2}\times P\left(1-P\right)}{e^{2}}\right]$$

Based on available data of 70% of total population in Singapore being Singaporeans and Permanent Residents (resident population) [32], 13% of resident population being elderly (65 years and above) [32] and close to 1 in 3 elderly aged between 65 and 74 years with grandchildren being involved in caregiving [20], the estimated range of grandparents/households to sample for this study, assuming a 50% response rate, was calculated as follows:

$$\begin{array}{l} \mathrm{Estimated}\;\mathrm{range}\;\mathrm{of}\;\mathrm{units}\;\mathrm{to}\;\mathrm{be}\;\mathrm{surveyed}=\mathrm{required}\;\mathrm{sample}\;\mathrm{size}\times \\ \frac{1}{\mathrm{proportion}\;\mathrm{of}\;\mathrm{resident}\;\mathrm{population}}\times \frac{1}{\mathrm{proportion}\;\mathrm{of}\;\mathrm{resident}\;\mathrm{population}\;\mathrm{being}\;\mathrm{elderly}}\times \frac{1}{\mathrm{predicted}\;\mathrm{response}\;\mathrm{rate}}\\ =385\times \frac{1}{0.7}\times \frac{1}{0.13}\times \frac{1}{0.5}\\ =(8462,\;8462\text{×}3)\\ =(8462,\;25,386) \end{array}$$

where 8462 is the lower limit of the number of units to be surveyed and 25,386 is the upper limit of the number of units to be surveyed after factoring in “1 in 3 elderly aged between 65 and 74 years with grandchildren were involved in caregiving.”

Bedok was chosen as the study’s sample location as it has the highest absolute elderly population [32] with several preschools and primary schools in the neighborhood. It also met the minimum number of households to be sampled. This increases the likelihood of finding suitable grandparents that fit the study’s inclusion criteria. Housing development board (HDB) blocks with high numbers of rental units were excluded as rental units may overrepresent lower income families and hence be unrepresentative of the target population given the eligibility criteria for such housing [33]. In addition, only a small proportion of elderly live in such units [20].

#### 2.3.3. Procedure

Prior to the recruitment process, an official notification letter regarding the upcoming study was distributed to the residents living in HDB flats in Bedok North, the chosen sample location. The interview-administered questionnaires were conducted on weekdays over a two-week period in February 2018. Individuals were approached door-to-door and only those who fulfilled the inclusion criteria and gave informed verbal consent using a participant information sheet were recruited.

Each participant was assigned a study number and no personal identifiers were collected to ensure anonymity. A maximum of two subjects per unit was surveyed. In the event that the household has more than two eligible subjects present, the aforementioned subjects were randomly numbered and two subjects were picked by pre-assigned numbers obtained from a random number generator. The questionnaires were administered by trained research team members in a language that both the participant and interviewer were competent in. Responses were recorded on a secure electronic platform. Translators were recruited to conduct the questionnaires in the relevant languages under the supervision of a research team member.

#### 2.3.4. Data Analysis

Descriptive statistics of various aspects of knowledge, attitudes, and practices were calculated and presented as percentages. For bivariate analyses, medians between groups were compared via Mann–Whitney U test and Kruskal–Wallis H test for 2 and >2 groups respectively. Chi-squared test of independence and chi-squared trend test were used to compare nominal and ordinal variables respectively. A *p*-value of <0.05 was regarded as statistically significant. IBM^®^ SPSS^®^ Statistics Version 24.0 was used for all data analyses.

## 3. Results

### 3.1. Qualitative Results and Analysis

The thematic analysis delineated various sub-themes under the three themes relating to the diverse aspects of grandparents’ influence on eating habits of grandchildren—knowledge, attitudes, and practices. Regarding knowledge, participants demonstrated awareness of various types of healthy and unhealthy food as well as the impact of feeding on the health of their grandchild. For attitudes, they expressed various feelings and thoughts regarding their feeding role and the feeding practices of other caregivers. With respect to practices, they reported influence over their grandchild’s eating habits via two main ways—direct influence and indirect influence. The pertinent themes, sub-themes, and codes are presented in Table 2.

### 3.2. Quantitative Results and Analysis

#### 3.2.1. Participant Demographics

In total 9105 households were knocked upon, of which 1025 units were eligible. A total of 396 participants (65% female, 35% male) were recruited out of the 1025 eligible households surveyed (1–2 per household), giving an overall response rate of 38.6%. Of the 1025 eligible units, 478 were unwilling to participate in the survey, while 151 were unable to participate due to scheduling difficulties. The mean age of the participants was 70.0 ± 7.4 years, with the majority (81.3%) being Chinese and the remaining being Malay, Indian, and Others. About half (46.7%) had an educational level of primary school and below. Detailed sample demographics are presented in Table 3.

Participants spent a median of 16 (IQR = 4, 30) days per month with their grandchildren, with 137 (35%) indicating that they were the primary caregivers. Of these, 105 (77%) were female (*p* < 0.001). A positive correlation was found between being a primary caregiver and time spent with the grandchild (*p* < 0.001) as well as living with the grandchild (*p* < 0.001). This corresponds to the definition of a primary caregiver as mentioned earlier. This suggests that time spent with the grandchild and/or living with the grandchild is mostly associated with being the primary caregiver. As such, instead of using all three variables, being a primary caregiver was logically chosen as the main independent variable.

#### 3.2.2. Assessment of Healthy Eating Knowledge

**Figure 1:**
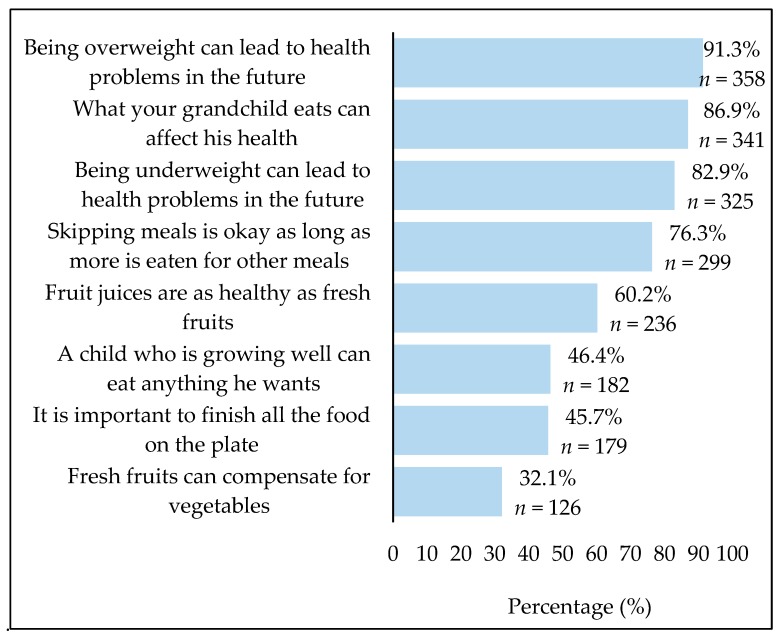
Percentage of correct responses to questions on knowledge of healthy eating and its impact on child health.

Out of 8 true-false knowledge questions, the median knowledge score was 5 (IQR = 4, 6). Participants generally demonstrated good knowledge of the impact of a child’s diet and weight on his health (Figure 1). A larger proportion of participants (91.3%) believed that being overweight will lead to future health problems compared to being underweight (82.9%) (McNemar *p* = 0.001). Several misconceptions were also found. These were centered around knowledge of healthy feeding practices. For example, many participants incorrectly answered that fruits can adequately replace vegetables in a child’s diet (67.9%), a child should finish all the food on the plate (54.3%), and should be allowed to eat anything he wants as long as he is growing well (53.6%)

It was found that less educated grandparents scored lower on the knowledge questions (*p* < 0.001) and cited more difficulties in learning about healthy eating (*p* = 0.006). Grandparents of Malay ethnicity were also found to have lower knowledge scores compared to the other races (*p* = 0.009). However, they were not found to have lower educational qualification levels compared to the other races. It is possible that other factors such as cultural differences account for this discrepancy between knowledge scores and educational background. More studies are needed to confirm this finding. No differences in knowledge scores were observed for gender of grandparent, age of grandchild, and whether the grandparent was the primary caregiver or not (Table 4).

Regarding learning about healthy eating, only slightly more than half (58.6%) of the participants cited no difficulties in doing so. Of those who did encounter difficulties, difficulty discerning between true and false information (44.5%), feeling overwhelmed by the amount of information available (43.3%), and not being able to keep up with available information (41.5%) were the most common challenges.

#### 3.2.3. Attitudes

Participants were questioned on their attitudes toward their feeding role as well as toward the feeding practices of the grandchild’s other caregivers using a five-point Likert scale. With regard to their attitudes toward their feeding role, almost half (47.7%) of the participants strongly agreed or agreed that they play an important role in feeding their grandchildren (data not shown in tables). Primary caregivers (*p* < 0.001), female (*p* < 0.001), Malay (*p* = 0.033), and grandparents younger than 70 years old (*p* < 0.001) believed they played an important role in feeding their grandchild (Table 5).

Many (64.6%) felt that the feeding practices of the grandchild’s other caregivers were mostly very healthy or healthy. With regard to strictness of the other caregivers’ practices, their responses were almost equally distributed among strict, neutral, and lenient.

#### 3.2.4. Feeding Practices

Regarding meal-time conditions, the majority (75.8%) of participants required their grandchild to eat together with the family sometimes or all the time. However, half of the grandparents (46.9%) reported allowing their grandchild to use electronic devices during meals sometimes or all the time (Table 6).

As part of the direct influence that participants had on their grandchild’s eating habits through preparing the type and amount of food served, the top three feeding activities engaged by participants sometimes or all the time were: Choose a wide variety of food for their grandchild (76.3%), cooking (66.1%), allowing their grandchild to decide how much food he/she eats (64.1%) (Table 6). A significant percentage (66.7%) rarely or never allowed their grandchild to eat unhealthy food, 76.6% choose a wide variety of food sometimes or all the time, and 47.2% rarely or never set a maximum limit on the amount of unhealthy food eaten. When asked how often limits were set for their grandchild’s food intake, more grandparents (38.8%) set a minimum limit compared to setting a maximum limit (29.2%) sometimes or all the time (McNemar *p* = 0.001).

In deciding what to feed their grandchild, the three most frequent considerations were: What they feel is healthy for their grandchild (59.6%); the grandchild’s preferences (58.3%); and what the other caregiver(s) wanted the child to eat (37.4%). Other considerations included: The grandchild’s appetite (32.8%); how the meal is prepared (15.9%); and the cost of the meal or ingredients (9.1%).

Slightly more than half of the participants experienced challenges when feeding their grandchild (59%). The top three difficulties encountered were the grandchild complaining he/she is too full (50.3%), the grandchild being playful and refuses to be fed (40.1%), and that the grandchild dislikes the type of food the participant gives him (38.7%). Other challenges encountered included: The grandchild has too much snacks between meals and refuses food (29.7%), dislikes the way food is prepared (13.4%), prefers to be fed by other caregivers (10.3%), conflict with other caregivers (10.3%), and difficulties in choosing healthier options (6.0%).

As part of the indirect influence that participants had on their grandchild’s eating habits through getting the grandchild to eat during meal times, the three most common practices employed to get their grandchild to obey them during feeding were: educating the child about the positive or negative effects of eating a particular food (51.0%); role-modelling (34.1%); and telling the grandchild that they will be rewarded (27.3%). Other methods employed included: telling him/her that their caregiver (apart from the grandparent) said so (26.0%), grandparent allows grandchild to do what he/she wants (23.4%), and warning grandchild that he/she will be punished (22.5%).

#### 3.2.5. Correlating Demographics with Feeding Practices

Differences in certain feeding practices were found between grandparents of varying demographics (Table 7). Grandparents who chose a wide variety of food were females (*p* = 0.006) or primary caregivers (*p* = 0.003). Those who rarely or never allowed their grandchild to eat unhealthy food were Chinese (*p* = 0.002) or had younger grandchildren (*p* = 0.005). Those who set a maximum limit on the amount of unhealthy food all the time or sometimes were female (*p* = 0.004), younger than 70 years old (*p* = 0.002), or the primary caregiver (*p* < 0.001). Highest educational qualification level did not correlate significantly with feeding practices (*p* > 0.05).

#### 3.2.6. Correlating Knowledge and Attitudes with Feeding Practices

Higher knowledge scores about healthy eating were significantly associated with sometimes or all the time choosing a wide variety of food (*p* = 0.003) and setting a maximum limit to the amount of unhealthy food (*p* = 0.001) (Table 8). However, there were no significant association between the participants’ knowledge scores and allowing grandchild to eat unhealthy food (*p* = 0.485).

Grandparents who viewed other caregivers’ feeding practices as healthy rarely or never allowed their grandchild to eat unhealthy food (*p* = 0.002). Participants who agreed or strongly agreed that they played an important role in feeding their grandchild were found to choose a wide variety of food (*p* < 0.001) and set a maximum limit to the amount of unhealthy food (*p* < 0.001) their grandchild eats. (Table 8).

#### 3.2.7. Sources of Influence on Grandparents

From a specified list, participants identified their personal experience (84.5%), media (55.8%) and the local Ministry of Health’s educational materials on healthy eating (36.8%) as the three most common sources that influence their views on healthy eating for their grandchild.

## 4. Discussion

This study’s findings highlight Singaporean grandparents’ knowledge, attitudes, and practices on the feeding and nutrition of their grandchildren.

### 4.1. Knowledge of Healthy Eating and Nutrition

Singaporean grandparents generally recognized that being overweight in childhood can lead to health problems later in life. This study showed that an overwhelming majority (91.3%) recognized that being overweight can lead to health problems for the grandchild in future. In contrast, studies in the United States [15] and China [8,9,10] showed trends of grandparents preferring obese children and believing that being heavy was associated with good nutrition. However, Singaporean grandparents, like their Chinese counterparts, still lack knowledge on healthy feeding practices, as evidenced by their median knowledge score of 62.5% (answering 5 out of 8 questions correctly) and food knowledge misconceptions such as the need to have the grandchild finish all the food on his plate. The lack of knowledge on healthy feeding could be multifactorial, of which lower education levels were identified as a significant associated factor. These persistent misconceptions can potentially contribute to feeding practices associated with obesity. Raising awareness of healthy feeding practices among grandparents thus remains a pressing issue to address.

### 4.2. Grandparents’ Attitudes toward Their Feeding Role

Only half of the grandparents in Singapore acknowledge the importance of their role in determining the nutrition of their grandchildren. Given the extent of their involvement identified in the analysis of feeding practices, this finding suggests that more should be done to allow grandparents to recognize the importance of their role in determining the nutrition and health of their grandchildren. This concept of grandparental responsibility for children’s feeding and nutrition is an important impetus which empowers the grandparent to make constructive changes for the benefit of their grandchild [9]. This is similar to American grandparents who cited the concept of parental responsibility for children’s eating, exercise habits, and body weight [15]. Likewise, grandparents in China expressed the desire to do a good job in fostering the child, including feeding the child well [11]. The sense of duty toward their grandchildren’s eating practices is relevant to consider for future interventions.

### 4.3. Grandparents’ Feeding Practices

The results suggest that grandparents in Singapore have significant influence on the feeding and nutrition of their grandchildren, both directly through food handling and indirectly through behavior modelling. This is especially important as about one-third of the Singaporean grandparents are primary caregivers for their grandchildren. Regarding direct feeding practices, a large proportion of grandparents surveyed (>68.5%) plan meals and/or cook for their grandchild, suggesting heavy involvement in determining the quality of food for their grandchild. This corresponds to international studies showing grandparents influenced their grandchild’s diet through food preparation [8,9,13,15].

When deciding what to feed their grandchild, similar to studies done in China [9] and the United States [15], Singaporean grandparents considered their grandchild’s food preferences and what they felt was healthy for them. Chinese grandparents cooked meals in line with what they believed was good and nutritious for the whole family and bought food according to their grandchild’s preferences [9]. African American grandmothers made healthier food choices for their grandchildren for disease prevention [34].

While the study shows that 60% of Singaporean grandparents make food decisions based on what they felt is healthy for their grandchild, some (33%) do allow their grandchild to eat unhealthy food occasionally. This is similar to Chinese and Australian grandparents. In the China study [9], where most grandparents were their grandchild’s primary caregiver, they were found to give snacks as a reward to control their grandchild’s behavior. In the Australian study [13], where grandparents were often not the primary caregiver, they indulged their grandchild in such unhealthy food as an act of doting on their grandchild. 

As part of indirect practice, educating their grandchild about healthy eating was the most common method used to get the grandchild to obey mealtime instructions. While the study did not shed light on the possible reasons for this finding, this can be explained by a study which found that many Singaporean grandparents take upon themselves to be educators for the younger generation by transmitting moral values, traditions, and beliefs [17]. This practice was also prevalent among grandparents in New Zealand [35] as they felt sharing of knowledge will help the grandchild to understand the significance of healthy eating and also help encourage them to engage in healthy eating habits.

### 4.4. Factors Associated with Grandparent Feeding Practices

This study demonstrated that grandparents’ demographics, knowledge, and attitudes are all associated with their practices.

Primary caregivers were found to be associated with choosing a wide variety of food and setting a maximum limit to the amount of unhealthy food. However, there was no significant association between primary caregivers and disallowance of unhealthy food. This may reflect a conflict faced by grandparents who are primary caregivers in which they have to maintain a parental figure by making healthy food choices as part of their responsibility, yet at the same time wanting to indulge their grandchildren. Such challenges are shared among grandparents overseas. A British study found that grandparents who spent more time caring for their grandchildren provided a healthier food and eating environment, but they also are more likely to use food as a reward [36]. Similarly in an Australian study, many grandparents who were their grandchildren’s primary caregiver expressed this role-identity conflict between a presumed grandparent role of acting as a friend or confidant and the reality of the grandparent-as-parent role as disciplinarians [37].

Better grandparent knowledge about healthy eating and its impact on child health was found to be correlated to healthier feeding practices, as higher knowledge scores were associated with setting a maximum limit on unhealthy food and giving the grandchild a wide variety of food. A Japanese study showed that nutritional knowledge of the caregivers contributes to healthier eating practices of the children [38], which is consistent with the findings of this study.

However, it was found that disallowing unhealthy food was not significantly associated with better knowledge. Awareness of healthy eating may not always translate into healthy feeding practices. Studies have shown that grandparents, despite having the knowledge, may still fail to recognize or discuss their grandchildren’s health problems [15], and continue to use food as a way to dote on them [13].

Of note, grandparents tend to set a minimum limit more than a maximum limit for the amount of food their grandchild eats. This could be in line with the evolutionary mismatch hypothesis [39]; despite the abundance of food in modern day life, there is still an inherent drive to maximize food intake at every meal and conversely no evolutionary advantage or impetus in setting a maximum limit. This comes despite the fact that a greater proportion of grandparents recognize that being overweight is a problem in grandchildren compared to being underweight. There seems to be a knowledge–practice dissociation; although grandparents know obesity is an issue, their practices are not in line with their knowledge. Despite this fact, Singaporean grandparents still flair better than their counterparts in other parts of the world such as China in recognizing childhood obesity. Based on Chinese studies [8,9,10], Chinese grandparents often viewed an overweight child as being healthy and well cared for.

A similar knowledge–practice dissociation was seen in a study done in New Zealand, where several factors were hypothesized to be at play, chiefly upstream structural elements such as employment, income, cultural knowledge, and government policies related to welfare and pensions [36]. The psychologically perceived role of the grandparent may further contribute to this dissociation, especially if it involves the element of indulging the grandchild with food [13].

### 4.5. Strengths

This is the first study of its kind in Singapore. The exploratory nature of the study provides new insight into the impact of these important caregivers on grandchildren feeding. The mixed methods approach enhances the understanding of this topic in Singapore’s unique context.

### 4.6. Limitations—Qualitative Component

The small sample size may not have allowed maximum thematic saturation to be reached as there could still be additional codes generated from further interviews although no further sub-themes were obtained. Additionally, checks can be done to enhance the credibility [40,41] of the results such as member check.

### 4.7. Limitations—Quantitative Component

There was little representation of employed grandparents and those living in private estates in this study due to methodological constraints. Future studies can consider including these groups of grandparents. Poor door opening rates and the fact that the survey was carried out within school and working hours also contributed to low response rates, limiting the available data. Being a self-reported study, response bias is inherently present and difficult to identify. Participants may feel obliged to give a socially acceptable answer even if they have differing views or practices. This was addressed by assuring grandparents of the anonymity of their responses. Being an exploratory study, the questionnaire developed was not evaluated for its reliability and validity. However, expert input, pilot and review was done before implementation. Further studies should consider validating the questionnaire prior to administration.

The cross-sectional nature of the questionnaire demonstrates prevalence but is unable to establish direction of results or cause and effect relationships. Further studies can thus consider adopting cohort or case control methodologies. Due to the cultural context of the study within Singapore, results are also non-generalizable to other international communities.

Additionally, while childhood obesity set the context of this study, objective measures of the grandchildren’s health status such as body-mass index were not obtained. Differences in their grandchildren’s weight could have explained the variation in certain practices among grandparents. For example, if they were underweight or normal weight, the grandparents might have felt more comfortable in not setting maximum amounts of unhealthy food or in allowing unhealthy food to be eaten, compared to a situation where the grandchild was obese. Future studies should consider correlating the grandchildren’s body mass index to grandparents’ KAP.

### 4.8. Implications for Research and Practice

Grandparents in Singapore, especially primary caregivers of grandchildren, have an important but often neglected influence of dietary habits of children. They are an essential target population for future policies combating childhood obesity. These findings suggest that improving grandparents’ feeding knowledge and practices can possibly be a valuable approach to healthier diets for children.

First, as many grandparents reference the media and the local Ministry of Health’s materials when obtaining information on healthy eating, health promotion organizations could consider education on multimedia platforms to improve grandparents’ knowledge of nutrition as well as courses to build practical skills for healthy feeding. Educational platforms include television, radio, and online social media programs and advertisements. As cooking was the most common activity as part of direct influence among grandparents, cooking classes along with the distribution of booklets with healthy recipes can be held for grandparents to expose them to healthy cooking and feeding practices for their grandchildren. Leveraging on grandparents’ concern about childhood obesity, misconceptions contributing to obesity can be highlighted to grandparents. Recognizing the diverse educational backgrounds, learning capabilities, and social set-ups of grandparents, such educational efforts may require a diverse spectrum of media and venues to ensure adequate outreach. This may also require support from the grassroots to promote awareness locally. Grassroots is an organization run by volunteers which helps organize a wide range of programs for residents, encourage community involvement, and raise awareness about community issues.

Second, as this study has found that grandparents most often consider what they feel is healthy for their grandchildren when deciding what to feed them, measures can be taken to assist them in this decision-making process during grocery shopping. Healthier food choice labelling may allow grandparents to easily differentiate healthy and unhealthy food. By making healthier food accessible and affordable for most grandparents, it overcomes barriers to motivate them to make healthier food choices.

This study demonstrated the influence of grandparents on grandchildren’s feeding habits. However, quantification of the influence will require correlating objective measures of the grandchild’s health status such as body-mass index to grandparents’ KAP. This can be a target for future studies to determine the magnitude of grandparents’ role in contributing to a child’s eating habits and obesity among other factors.

Finally, progressive policies targeting grandparents’ feeding practices and grandparents’ evolving knowledge and attitudes will precipitate shifts in how children are fed. Follow-up studies assessing the grandparents’ KAP should thus be administered to assess for such trends. This allows evaluation of the efficacy and success of interventions which can then be improved further. Such continuous efforts help generate the change in children’s eating habits necessary to tackle childhood obesity.

## 5. Conclusions

Grandparents are an important but often neglected influence of dietary habits of children. This study explores the views and practices of Singaporean grandparents with regard to feeding their grandchildren, identifying grandparents as a significant target population for improving children’s food practices. With the pressing prevalence of childhood obesity in Singapore and worldwide, the influence of grandparents’ feeding practices on this phenomenon merit consideration in future studies and in formulation of healthcare policies tackling childhood obesity.

## Figures and Tables

**Table 1:** Main questions of the interview guide.

	To Assess:	Questions
	Screening questions to establish participant’s profile and assess if participants fulfil the inclusion criteria.	Do you have a grandchild? How old is your grandchild? On average, how many days do you physically spend with your grandchild per month?
**Topic 1**	The presence of influence of grandparents on the eating habits of their grandchildren. How grandparents influence the eating habits of their grandchildren. Their attitudes regarding their role in feeding their grandchildren.	In what ways do you influence your grandchild’s eating habits?
	Grandparents’ involvement in meal planning and determining quantity and quality of food. Methods used to get their grandchildren to eat as they have planned.	Follow-up questions ^1^: How do you influence the meal planning for your grandchild? How do you control how much food your grandchild eats? How do you encourage/discourage your grandchild to eat as you planned?
**Topic 2**	Knowledge of healthy eating regarding their grandchildren.	What do you think you should feed your grandchild? What do you think you should not feed your grandchild?
	Importance of healthy eating to grandparents.	Follow-up questions: How important is healthy eating with regard to your grandchild?

^1^ Follow-up questions are probes used as an extension of the main question(s) within the topic.

**Table 2:** How analysis of interviews guided questionnaire crafting.

Themes	Sub-themes	Codes	Example Question from Interviewer-Administered Questionnaire
**Knowledge**	Knowledge of the food itself	Views on type of food (positive)	I2: The amount of fruit juices children drink is not a major concern as fruit juices are as healthy as fresh fruits. I3: If your grandchild does not like vegetables, he/she should take more fruits to compensate instead.
		Views on type of food (negative)	
		Views on regularity of meals	I5: It is okay for your grandchild to skip meals if he/she eats more for other meals.
		Views on quantity of food	I4: As long as your grandchild is growing well, it is okay to allow him/her to eat anything he/she wants. I6: It is important for your grandchild to finish all the food on his/her plate.
		Views on food preparation	-
	Knowledge of the impact of feeding	Health consequences (positive)	-
		Health consequences (negative)	I7: If your grandchild is overweight, he/she is likely to have health problems in the future. I8: If your grandchild is underweight, he/she is likely to have health problems in the future.
		Importance of healthy eating	I1: What your grandchild eats can affect his/her health.
	Factors affecting knowledge	Factors that influence views on feeding	J1: What influences your views on healthy eating for your grandchild? Your parents/elders/traditional beliefs. Your friends. Your children. Your grandchild. Your doctor/nutritionist. Personal experience. Ministry of Health’s promotion of health and nutrition (TV, posters, leaflets, and pamphlets in polyclinics and hospitals). Media (e.g., newspapers, books, TV, Internet, radio)
**Attitudes**	Attitude toward feeding role		H2. You play an important role in feeding your grandchild.
	Attitude toward parents’ feeding practices		H1. How do you feel about the other caregivers’ feeding practices? (a) Scale from very healthy to very unhealthy. (b) Scale from very strict to very lenient.
	Attitude toward feeding challenges		-
**Practices**	Direct influence [9] ^1^	Determining type of food	D1(a). How often are you involved in planning what your grandchild has for meals/snacks? D2. How often do you allow your grandchild to eat unhealthy food (e.g., sweets/soft drinks/salty snacks/high fat foods/fast food)? D3. How often do you choose a wide variety of food (food from various food groups e.g., meat, fruits, and vegetables, grains) for your grandchild to eat?
		Determining quantity of food	D1(b). How often are you involved in planning how much your grandchild has for meals/snacks? D4. How often do you allow your grandchild to decide how much food he/she eats? D5. How often do you set a minimum amount of food your grandchild eats during mealtimes? D6. How often do you set a maximum limit to the amount of food your grandchild eats during mealtimes? D7. How often do you set a maximum limit to the amount of unhealthy food (e.g., sweets/sodas/salty snacks/high-fat foods/fast food)?
		Spoon-feeding	D1(f). How often are you involved in spoon-feeding your grandchild?
		Preparation of meals	D1(c). How often are you involved in cooking for your grandchild’s meals?
	Indirect influence [9] ^2^	Reinforcement	E1. With regard to feeding your grandchild, you get him/her to obey your instructions by: (a) Telling him/her that he/she will get a reward. (c) Educating him/her about the positive or negative effects of eating a particular type/amount of food.
		Punishment	E1. With regard to feeding your grandchild, you get him/her to obey your instructions by: (b) Warning him/her that he/she will get punished.
		Neutral response	E1. With regard to feeding your grandchild, you get him/her to obey your instructions by: (f) You just let him do what he/she wants.
	Factors that influence feeding practices		F1. How do you decide what to feed your grandchild? (a) Your grandchild’s preference.(b) Your grandchild’s usual appetite/appetite at that point in time.(c) What you feel is healthy for your grandchild.(d) What your grandchild’s other caregiver(s) (e.g., parents, domestic helper) want your grandchild to eat? (e) How the meal is prepared (e.g., difficulty, time needed, ingredients you have left over at home)? (f) Cost of meal/ingredients.
	Challenges to feeding	Child’s negative receptiveness to feeding	G1. Do you have difficulties feeding your grandchild due to any of the following reasons ?(a) He/she says he/she is full. (b) He/she eats too much snacks between meal times and refuses his/her meals. (c) He/she does not like the type of food you give him/her (e.g., does not like meat, vegetables). (d) He/she does not like the way you prepare the food (e.g., too spicy, too little salt, too healthy as it is mostly steamed etc.,). (e) He/she prefers to be fed by other caregivers (e.g., parents, domestic helper etc.,). (f) He/she is very playful and does not let you feed him/her (e.g., running around, watching television).
		Child’s positive receptiveness to feeding	G1. Do you have difficulties feeding your grandchild due to any of the following reasons? (i) You have no difficulties in doing so.
		Conflict between caregivers	G1. Do you have difficulties feeding your grandchild due to any of the following reasons? (g) You have conflicts with other caregivers regarding how to feed your grandchild.
	Meal conditions	Meal conditions	C1. When you are with your grandchild, how often do you get him/her to eat together with the rest of the family (regardless of the type of meal, including snacks and fruits)? C2. How often do you allow your grandchild to use electronic devices (e.g., television/tablet/phone) during meals ?C3. On average, you prepare home-cooked meals for your grandchild ___ times a week. C4. On average, you obtain meals from outside (includes both eating out and takeaways) for your grandchild ___ times a week.

^1^ The impact on their grandchildren’s eating habits through the type and amount of food served. ^2^ The impact on their grandchildren’s eating habits through education and role modelling.

**Table 3:** Demographics of respondents who completed the quantitative questionnaire (*n* = 396).

**Characteristics**	**N (%)**
Gender	
Male	138 (34.8)
Female	258 (65.2)
Race	
Chinese	322 (81.3)
Malay	55 (13.9)
Indian	17 (4.3)
Others	2 (0.5)
Highest educational qualification attained ^1^	
Primary school and below	182 (46.7)
Secondary school	150 (38.5)
Post-secondary (A-level, diploma, ITE)	46 (11.8)
University degree and above	12 (3.1)
Lives with grandchild ^2^	123 (31.2)
Primary caregiver (the person who spends the most time with the grandchild and performs most of the caregiving tasks)	137 (34.6)
**Characteristics**	**Median (IQR)**
Age of grandparent (years) ^2^	70 (65, 74)
Age (years) of the one grandchild that they spend the most time with	7 (3, 9)
Days of encounter with grandchild per month	16 (4, 30)

^1^*n* = 390. ^2^ *n* = 394.

**Table 4:** Comparison of knowledge scores amongst different demographic groups (*n* = 396) *.

Characteristics	Median (IQR)	*p*-Value
Gender		0.419
Male	5 (4, 6)	
Female	5 (4, 6)	
Race		0.009
Chinese	5 (4, 6)	
Malay	5 (4, 6)	
Indian	5 (4, 6)	
Others	4 (4, 5)	
Highest Qualification Attained		<0.001
Primary school and below	5 (4, 6)	
Secondary school	5 (4, 6)	
Post-secondary (A-level, diploma, ITE)	6 (5, 7)	
University degree and above	7 (6, 7)	
Age of grandparent (years)		0.018
<70	6 (4, 7)	
≥70	5 (4, 6)	
Age of grandchild (years)		0.524
0–1	5 (4, 6)	
2–6	5 (4, 6)	
7–9	5 (4, 7)	
10–12	5 (4, 6)	
Primary caregiver		0.883
Yes	5 (4, 6)	
No	5 (4, 6)	

* Difference in scores between groups was compared via the Mann–Whitney U Test and Kruskal–Wallis H Test for 2 categories and >2 categories respectively.

**Table 5:** Comparison of attitude toward own feeding role among different demographic groups (*n* = 396) (%) *.

Characteristic	You Play an Important Role in Feeding Your Grandchild.			*p*-Value
	Strongly Disagree/Disagree	Neutral	Strongly Agree/Agree	
Gender				<0.001
Male	51.5	16.7	31.9	
Female	28.7	15.1	56.2	
Race				0.033
Chinese	38.2	16.8	45	
Malay	27.3	5.5	67.3	
Indian	38.2	23.5	41.2	
Others	50	50	0	
Highest Qualification Attained **				0.83
1	36.8	17	46.2	
2	35.3	15.3	49.3	
3	43.5	13	43.5	
4	25	16.7	58.3	
Age of grandparent (years)				<0.001
<70	25	16.3	58.7	
≥70	46.7	15.2	38.1	
Age of grandchild (years)				0.651
0–1	45.7	17.1	37.1	
2–6	33.1	15.6	51.3	
7–9	33.9	15.6	50.5	
10–12	42.4	15.2	42.7	
Primary caregiver				<0.001
Yes	18.2	16.1	65.7	
No	46.3	15.4	38.2	

* % Percentage of participants within the demographic category that indicated the option. $\chi^{2}$ test of independence and $\chi^{2}$ trend test was performed for nominal and ordinal demographic variables respectively. ** (1) Primary school and below; (2) secondary school; (3) post-secondary (A-level, diploma, ITE); (4) university degree and above.

**Table 6:** Grandparents’ feeding practices (*n* = 396) (%) *.

Question	All the Time/ Sometimes	Rarely/Never
**Meal Condition**		
Grandchild eats together with the rest of the family	75.8	24.3
Grandchild is allowed to use electronic devices during meals	46.9	53.2
**Direct Influence** **How Often Are You Involved**		
In choosing a wide variety of food for the grandchild to eat	76.3	23.7
In cooking for the grandchild’s meals	66.1	33.9
In allowing the grandchild to decide how much food he/she eats	64.1	36.0
In planning what the grandchild has for meals/snacks	60.4	39.7
In deciding when the grandchild has his/her meals	55.4	44.5
In setting a maximum amount of unhealthy food for the grandchild	52.9	47.2
In planning how much the grandchild has for meals/snacks	52.2	47.7
In setting a minimum amount of food for the grandchild	38.8	61.2
In spoon-feeding the grandchild	34.2	65.8
In allowing your grandchild to eat unhealthy food	33.3	66.7
In setting a maximum amount of food for the grandchild	29.2	70.8
In presenting/preparing the food such that it is more interesting/appealing	29.0	71.0

* % refers to the percentage of respondents who indicated that particular answer for the question. Row percentages may not add up to 100.0 due to the rounding off to 1 decimal place. “Not Applicable” responses were excluded from the analysis.

**Table 7:** Comparison of feeding practices amongst different demographic groups (*n* = 396) *.

Characteristic	Choose a Wide Variety of Food for Grandchild	Allow Grandchild to Eat Unhealthy Food	Set a Maximum Limit of Unhealthy Food Grandchild Takes
	Sometimes/All the Time	Rarely/Never	Sometimes/All the Time
Gender			
Male	70.00%	60.90%	41.90%
Female	80.60%	69.80%	58.30%
*p-*value	0.006	0.084	0.004
Race			
Chinese	76.80%	70.10%	52.30%
Malay	72.30%	57.70%	58.80%
Indian	85.70%	26.70%	42.90%
Others	0.00%	100.00%	50.00%
*p-*value	0.226	0.002	0.721
Age of grandparent (years)			
<70	80.20%	68.50%	62.60%
≥70	73.60%	65.50%	45.60%
*p-*value	0.147	0.548	0.002
Age of grandchild (years)			
0–1	61.50%	91.70%	33.30%
2–6	78.50%	73.50%	55.20%
7–9	77.80%	52.40%	52.00%
10–12	75.60%	65.60%	54.50%
*p-*value	0.579	0.005	0.461
Primary caregiver			
Yes	85.50%	65.90%	68.60%
No	71.20%	67.20%	44.60%
*p-*value	0.003	0.795	<0.001

* % percentage in each demographic category indicating sometimes/all the time for the stated feeding practice. $\chi^{2}$ test of independence and $\chi^{2}$ trend test were performed for nominal and ordinal demographic variables respectively.

**Table 8:** Comparison of knowledge and attitudes with practices (*n* = 396) (N (%)) *.

Characteristic of Knowledge and Attitudes	Choose a Wide Variety of Food for Grandchild		Allow Grandchild to Eat Unhealthy Food		Set a Maximum Limit of Unhealthy Food Grandchild Takes	
	Rarely/Never	Sometimes/All the Time	Rarely/Never	Sometimes/All the Time	Rarely/Never	Sometimes/All the Time
Knowledge score						
Poor (0–3)	19 (42.2)	26 (57.8)	34 (73.9)	12 (26.1)	32 (69.6)	14 (30.4)
Average (4–5)	31 (22.3)	108 (77.7)	91 (59.9)	61 (40.1)	69 (46.9)	78 (53.1)
Good (6–8)	29 (18.4)	129 (81.6)	120 (71.4)	48 (28.6)	62 (40.0)	93 (60.0)
*p-*value	0.003		0.485		0.001	
Attitude toward other caregivers’ feeding practices						
Very healthy/healthy	47 (21.6)	171 (78.4)	169 (71.9)	66 (28.1)	94 (42.3)	128 (57.7)
Neutral	29 (30.9)	65 (69.1)	59 (60.2)	39 (39.8)	55 (57.9)	40 (42.1)
Very unhealthy/unhealthy	6 (18.2)	27 (81.8)	18 (50.0)	18 (50.0)	17 (50.0)	17 (50.0)
*p-*value	0.572		0.002		0.056	
Attitude toward other feeding practices						
Very strict/strict	28 (24.6)	86 (75.4)	88 (72.1)	34 (27.9)	48 (41.7)	67 (58.3)
Neutral	28 (22.4)	97 (77.6)	86 (64.2)	48 (35.8)	64 (50.0)	64 (50.0)
Very lenient/lenient	26 (24.5)	80 (75.5)	72 (63.7)	41 (36.3)	54 (50.0)	54 (50.0)
*p-*value	0.988		0.167		0.212	
“You play an important role in feeding your grandchild”						
Strongly agree/agree	29 (16.3)	149 (83.7)	120 (68.2)	56 (31.8)	62 (36.3)	109 (63.7)
Neutral	8 (15.1)	45 (84.9)	29 (53.7)	25 (46.3)	25 (47.2)	28 (52.8)
Strongly disagree/disagree	45 (39.1)	70 (60.9)	98 (70.0)	42 (30.0)	79 (61.7)	49 (38.3)
*p-*value	<0.001		0.807		<0.001	

* N(%) Count and row percentage of participants within the characteristic category that indicated the option; pooled responses of rarely and never indicated on the left sub-column, pooled responses of sometimes and all the time indicated on the right sub-column. As all the above were ordinal variables, χ^2^ trend test was performed for the above analysis.

## Supplementary Materials

The following are available online at https://www.mdpi.com/2072-6643/11/7/1696/s1, Supplementary file 1: Questionnaire.
