# Supplementary material for: Feeding-Related Knowledge, Attitudes, and Practices among Grandparents in Singapore
Source: Nutrients. 2019 Jul 23;11(7):1696. doi: 10.3390/nu11071696 (PMC6683024; doi:10.3390/nu11071696)
Supplement: Supplementary file 1 [file nutrients-11-01696-s001.pdf]

QUESTIONNAIRE

Project title:
Grandparents' Knowledge, Attitudes and Practices on the Feeding of Grandchildren in Singapore

Participant ID: ____-____-____

Questions:

SECTION A: Screening questions

1. Do you have a grandchild?
   a. Yes
   b. No
2. How old is your grandchild? ____ *(rounded off to the nearest year) (Should be <u><</u>12)*
   Note: If you have more than 1 grandchild, please pick 1 grandchild that you think you spend the most time with.
3. On average, how many days do you physically spend time with your grandchild per month (regardless of no. of hours per day)? ____days per month *(min:2; max: 31)*
4. Select your nationality: (*Has to be Singaporean/PR*)
   a. Singaporean/PR
   b. Non-Singaporean/ Non-PR

SECTION B: Demographics

1. What is your age? ______ *(in years)*
2. Select your gender:
   a. Male
   b. Female
3. Select your race:
   a. Chinese
   b. Malay
   c. Indian
   d. Others, please specify: ____________
4. Select your highest education level:
   a. Primary school and below
   b. Secondary school
   c. Post-secondary (A level, diploma, ITE)
   d. University degree and above
5. Do you live with your grandchild?

---

a. Yes
b. No

6. Are you the primary caregiver for your grandchild (person who spends most time with the grandchild and performs most of the caregiving tasks)?
a. Yes
b. No

GRANDPARENTS' FEEDING PRACTICES

SECTION C: Meal Condition

Please indicate the frequency for questions 1 and 2 only.

(a) All the time	*(b) Sometimes*	*(c) Rarely*	*(d) Never*	*(e) Not applicable, please specify*

A. Family

1.When you are with your grandchild, how often do you get him/her to eat together with the rest of the family (regardless of the type of meal, including snacks and fruits)? ____

B. Entertainment

2. How often do you allow your grandchild to use electronic devices (e.g. television / tablet / phone) during meals? ____

C. Location *(you do not need to indicate frequency)*

3. On average, you prepare home-cooked meals for your grandchild ___ times a week

4. On average, you obtain meals from outside (includes both eating out and takeaways) for your grandchild ___ times a week

SECTION D: Direct Influence

Please indicate frequency for all the following questions:

(a) All the time	*(b) Sometimes*	*(c) Rarely*	*(d) Never*	*(e) Not applicable, please specify*

1. How often are you involved in?:
a. Planning what your grandchild has for meals/snacks ____
b. Planning how much your grandchild has for meals/snacks ____
c. Cooking for your grandchild's meals ____
d. Presenting/preparing the food such that it is more interesting/appealing (e.g. cut ingredients into various shapes/small pieces) so that your grandchild will eat. ____
e. Deciding when your grandchild has his/her meals ____
f. Spoonfeeding your grandchild ____

Determining the type of food given

2. How often do you allow your grandchild to eat unhealthy food (e.g. sweets/soft drinks/ salty snacks/high fat foods/fast food)? ____

3. How often do you choose a wide variety of food (food from various food groups e.g. meat, fruits and vegetables, grains) for your grandchild to eat? ____

Determining the quantity of food given

4. How often do you allow your grandchild to decide how much food he/she eats? ____

5. How often do you set a minimum amount of food your grandchild eats during mealtimes? ____

6. How often do you set a maximum limit to the amount of food your grandchild eats during mealtimes? ____

7. How often do you set a maximum limit to the amount of unhealthy food (e.g. sweets/sodas/salty snacks/high fat foods/fast food) your grandchild eats? ____

SECTION E: Indirect influence

1. With regards to feeding your grandchild, you get your him/her to obey your instructions by: (*You may select more than one option)*
   a. Telling him/her that he/she will get a reward
   b. Warning him/her that he/she will get punished
   c. Educating him/her about the positive or negative effects of eating a particular type/amount of food
   d. Telling him/her their caregiver (other than yourself) said so.
   e. Doing what you want him/her to do and telling him/her to do the same (being a role model to your grandchild)
   f. You just let him do what he/she wants
   g. Others (please specify): ____
   ____
   ____
   ____

SECTION F: Factors that influence Feeding Practices

1. How do you decide what to feed your grandchild? *(You may select more than one option):*
   a. Your grandchild's preference
   b. Your grandchild's usual appetite/ appetite at that point in time
   c. What you feel is healthy for your grandchild

d. What your grandchild's other caregiver(s) (e.g. parents, domestic helper) want your grandchild to eat
e. How the meal is prepared (e.g. difficulty, time needed, ingredients you have left over at home)
f. Cost of meal / ingredients
g. Others (please specify):

____

____

____

____

<u>SECTION G: Feeding challenges</u>

1. Do you have difficulties feeding your grandchild due to any of the following reasons? *(You may indicate more than one option)*

a. He/she says he/she is full
b. He/she eats too much snacks between meal times and refuses his/her meals
c. He/she does not like the type of food you give him/her (e.g. does not like meat, vegetables)
d. He/she does not like the way you prepare the food (eg. too spicy, too little salt， too healthy as it is mostly steamed etc)
e. He/she prefers to be fed by other caregivers (eg. parents, domestic helper etc)
f. He/she is very playful and does not let you feed him/her (e.g. running around, watching television)
g. You have conflicts with other caregivers regarding how to feed your grandchild
h. You have difficulties in choosing healthier options for your grandchild
i. You have no difficulties in doing so.
j. Others (please specify):

____

____

____

____

<u>ATTITUDES</u>

<u>SECTION H: Attitudes</u>

<u>Attitudes toward other caregiver's feeding practices</u>

1. How do you feel about the other caregivers' feeding practices (*Please only indicate 1 option for questions 1a and 1b)*?

a.

(a) Very healthy	(b) Healthy	(c) Neutral	(d) Unhealthy	(e) Very unhealthy

b.

(a) Very strict	(b) Strict	(c) Neutral	(d) Lenient	(e) Very lenient

<u>Attitudes towards feeding role</u>

2. You play an important role in feeding your grandchild *(Please only indicate 1 option)*

(a) Strongly agree	*(b) Agree*	*(c) Neutral*	*(d) Disagree*	*(e) Strongly Disagree*

<u>PERCEPTION AND KNOWLEDGE</u>

<u>SECTION I: Knowledge on Nutrition</u>

Please indicate your opinion on each of the following statements:

(a) True	*(b) False*

1. What your grandchild eats can affect his/her health. ____
2. The amount of fruit juices children drink is not a major concern as fruit juices are as healthy as fresh fruits. ____
3. If your grandchild does not like vegetables, he/she should take more fruits to compensate instead. ____
4. As long as your grandchild is growing well, it is okay to allow him/her to eat anything he/she wants. ____
5. It is okay for your grandchild to skip meals if he/she eats more for other meals. ____
6. It is important for your grandchild to finish all the food on his/her plate. ____
7. If your grandchild is overweight, he/she is likely to have health problems in the future. ____
8. If your grandchild is underweight, he/she is likely to have health problems in the future. ____

<u>SECTION J: Factors influencing views on feeding</u>

1. What influences your views on healthy eating for your grandchild? (*You may select more than one option*)
   a. Your parents/elders/follow tradition
   b. Your friends
   c. Your children
   d. Your grandchild
   e. Your doctor/nutritionist

f. Personal experience
g. Ministry of Health's promotion of health and nutrition (TV, Posters, Leaflets and Pamphlets in Polyclinics and Hospitals)
h. Media (e.g. newspapers, books, TV, Internet, radio)
i. Others (please specify):
____
____
____
____

2. Do you have difficulties learning about healthy eating and nutrition due to any of the following reasons? (*You may select more than one option)*

a. The information is always changing and you cannot keep up.
b. You cannot understand all the information available
c. You do not have the interest to learn
d. You do not have access to learning materials
e. You feel overwhelmed by the amount of information available.
f. You have difficulty discerning which information is true and which is false
g. You have no difficulties in doing so.
h. Others (please specify):
____
____
____
